# Association of a polygenic risk score with low trauma fractures in people with HIV – The swiss HIV cohort study

**DOI:** 10.1371/journal.pone.0342748

**Published:** 2026-02-11

**Authors:** Claudio Strickler, Christian W. Thorball, Isabella C. Schoepf, Johannes Schwenke, Barbara Hasse, Lene Ryom, Maria C. Thurnheer, Christoph Fux, Christian R. Kahlert, Enos Bernasconi, Alexandra Calmy, Olivier Lamy, Huldrych F. Günthard, Bruno Ledergerber, Jacques Fellay, Philip E. Tarr, the Swiss HIV Cohort Study

**Affiliations:** 1 University Center Internal Medicine and Infectious Diseases Service, Kantonsspital Baselland, University of Basel, Bruderholz, Switzerland; 2 Precision Medicine Unit, Lausanne University Hospital and University of Lausanne, Lausanne, Switzerland; 3 School of Life Sciences, Ecole Polytechnique Fédérale de Lausanne, Lausanne, Switzerland; 4 Department of Visceral Surgery and Medicine, Inselspital, Bern University Hospital, University of Bern, Bern, Switzerland; 5 CLEAR Methods Center, Division of Clinical Epidemiology, Department of Clinical Research, University Hospital Basel, University of Basel, Basel, Switzerland; 6 Department of Infectious Diseases and Hospital Epidemiology, University Hospital Zurich, University of Zurich, Zurich, Switzerland; 7 CHIP, RIgshospitalet, University of Copenhagen, Copenhagen, Denmark; 8 Department of Clinical Medicine, University of Copenhagen, Copenhagen, Denmark; 9 Department of Infectious Diseases, Bern University Hospital, University of Bern, Bern, Switzerland; 10 Department of Infectious Diseases, Kantonsspital Aarau, Aarau, Switzerland; 11 Division of Infectious Diseases, Kantonsspital St. Gallen, St. Gallen , Switzerland; 12 Division of Infectious Diseases, Ente Ospedaliero Cantonale, Lugano, Switzerland; 13 University of Geneva, Geneva, Switzerland; 14 Università della Svizzera italiana, Lugano, Switzerland; 15 Division of Infectious Disease, Geneva University Hospital, Geneva, Switzerland; 16 Bone Unit, Lausanne University Hospital, University of Lausanne, Lausanne, Switzerland; 17 Institute of Medical Virology, University of Zurich, Zurich, Switzerland; Federal University of Minas Gerais: Universidade Federal de Minas Gerais, BRAZIL

## Abstract

**Background:**

Polygenic risk scores (PRS) are likely to enter routine clinical care for individual disease risk prediction in the next 10 years. We recently showed that the bone mineral density-associated gSOS-PRS is independently associated with a > 4-fold increased risk of osteoporosis in the Swiss HIV Cohort Study (SHCS). Here we investigate whether this PRS is also associated with low trauma fractures (LTF) in people with HIV in the SHCS.

**Methods:**

Applying a case-control design, cases had a first LTF (1994–2022) and LTF-free controls were matched on age, sex and observation time. We obtained univariable odds ratios (OR) for LTF in SHCS participants of European descent, based on a genome-wide PRS built from 9413 LTF-associated single nucleotide polymorphisms (SNPs). In multivariable analysis, LTF odds ratios of the PRS were adjusted for non-genetic (traditional and HIV-related) LTF risk factor profile including potentially adverse antiretroviral exposures.

**Results:**

We included 277 SHCS participants with a first LTF (cases) and 796 LTF-free controls (median age 55 years; 68% male; 91% with suppressed HIV RNA). Participants with the most unfavorable genetic background (top quintile of the gSOS-PRS) had univariable and multivariable LTF-OR of 2.30 (95% confidence interval, 1.49–3.56) and 2.30 (1.43–3.72), respectively, compared to participants with the most favorable genetic background (bottom gSOS-PRS quintile). Participants with the most unfavorable non-genetic risk factor profile (top quintile) had an adjusted gSOS-OR of 7.42 (95% confidence interval [CI], 4.3–12.82), compared with participants in the bottom quintile.

**Conclusions:**

In people with HIV in Switzerland, an unfavorable PRS was independently associated with LTF risk after adjustment for traditional and HIV-related LTF risk factors.

## Introduction

Bone health is a major long term concern in people living with HIV who have a considerably increased risk of osteoporosis and of low trauma fractures (LTF) compared to the general population [1,2]. The pathogenesis of osteoporosis and LTF in HIV is influenced by traditional risk factors (e.g., age, low body mass index, smoking and other substance use, corticosteroids) as well as HIV-associated factors including injection drug use, chronic hepatitis C, the effects of immunosuppression and of certain antiretroviral agents [3–7].

Individual LTF risk is also in part hereditary. Common single nucleotide polymorphisms (SNPs) that contribute to important diseases (e.g., osteoporosis, cardiovascular disease, diabetes) were first reported more than 30 years ago. Initial results, often based on small studies that investigated only a few candidate SNPs, frequently yielded false-positive results [8,9]. Approximately 15 years ago, genome-wide association studies (GWAS) became the standard in the field, and several large GWAS have identified common genetic variants that contribute to LTF risk in the general population [10–13]. In the past decade, genetic prediction of common diseases has now moved from single SNPs to the application of polygenic risk scores (PRS) [14–16]. Compared to previous approaches, individual PRS can substantially improve genetic prediction, by including up to several million common SNPs of smaller effect sizes in addition to individual or only genome-wide significant SNPs [15–17].

With one exception (HLA-B*57:01 testing to predict abacavir hypersensitivity [18]), genetic testing has not yet entered clinical HIV practice [19] but is predicted to be adopted in the coming years, based on recent studies documenting the predicting power of genetics for major clinical endpoints, advances in sequencing technology and significantly decreasing cost. For these reasons, we have now conducted several PRS studies in the Swiss HIV Cohort Study (SHCS) on the genetic associations with major aging-associated outcomes relevant to the unique setting of HIV, including chronic kidney disease [20,21], acute coronary artery disease events [14], subclinical coronary atherosclerosis [22], and osteoporosis [23]. The growing interest in genetic testing in clinical HIV care is driven by the potential to comprehensively predict a patient’s genetic risk across numerous clinically relevant endpoints for which robust genetic data are now available.

Regarding bone health in people with HIV (PWH), we recently found osteoporosis to be independently associated with the bone mineral density (BMD)-associated gSOS-PRS in 438 SHCS participants after adjustment for established traditional and HIV-associated risk factors [23]. Specifically, the 20% of PWH with the most unfavorable (top quintile) BMD-associated PRS had a more than 4-fold increased prevalence of osteoporosis on dual-energy X-ray absorptiometry (DXA) compared to the 20% of PWH with the most favorable (bottom quintile) PRS in multivariable-adjusted analyses [23]. Moreover, the LTF incidence in people with HIV in Switzerland is decreasing, both in SHCS participants with favorable and unfavorable genetic background [24].

In the present report, we provide an updated LTF prediction analysis in PWH in Switzerland, in extension of our GWAS study from 2016 which showed no evidence of an increased LTF risk among PWH who had an unfavorable genetic background [25]. However, our 2016 study was limited by sample size (103 PWH with LTF, 206 fracture-free controls), and by the genetic knowledge existing in 2014 (6 genome-wide significant SNPs associated with osteoporosis in the general population).

We now analyze 277 PWH with a first validated LTF and 796 controls, using a PRS built from >9000 validated, LTF-associated SNPs [10]. Because of the concern that aging in PWH may be accelerated and/or accentuated, we also investigated SNPs that have reliably associated with successful aging and longevity in large studies in the general population including LTF observational studies [26,27].

## Methods

### Study population

The research project was approved by the Cantonal Ethics Commission Zurich, Section B (Prof. em. Dr. med. Konrad E. Bloch) on 26th April 2024 (Application BASEC-no.: 2023–02080). Participants were enrolled in the Swiss HIV Cohort Study (SHCS, www.shcs.ch) and provided written informed consent for genetic testing [28]. We included participants of our previous LTF study published in 2016 (with validated LTF until 31.12.2014) [25], plus additional validated LTF cases 01.01.2015–31.12.2022 (study period 1.1.1994–31.12.2022). Because previous LTF-GWAS in the general population were conducted in populations of mostly European descent [11], the study was restricted to participants of self-reported European descent. Because PWH are at increased risk for osteoporosis and LTF compared to the general population, we limit this genetic analysis to the specific context of PWH.

### Low-Trauma Fractures

Each LTF was validated by the treating HIV physician and the main investigators (CS, ICS, PET) using a standardized form documenting the presence, date, body site, and low trauma nature of all fractures in the SHCS, as we reported previously [25]. LTFs were defined as resulting at age ≥ 18, at walking speed or less, and from a fall that occurred at standing height or less. Asymptomatic vertebral fractures detected by imaging were included, and diagnosis relied on the attending radiologist’s report using as fracture date the imaging date. Because fractures of the skull, cervical spine, fingers, and toes are typically considered traumatic in nature, these were excluded. To avoid including pathological fractures, all fractures occurring in persons with a history of malignancy were excluded.

### Case-control matching

As done previously [25], we aimed to select 3 controls who were LTF-free at the LTF date of the corresponding case (matching date) [29]. We used incidence density matching [30], i.e., we matched controls on similar observation *duration*, and their observation *period* was during similar calendar periods, in order to account for differences in ART in use at different times and other differences over time. We used as matching criteria sex at birth, age at registration + /- 5 years, and date of SHCS registration + /- 2 years. Cases were observed until the LTF date and controls were required to have a regular SHCS follow-up visit after the LTF date of their corresponding case, respectively. Observation time was calculated from registration until date of LTF for cases or date of LTF of the corresponding case for controls. We did not allow re-use of controls.

### Non-genetic LTF risk factors

As we reported previously [23,25], covariables included in the statistical models were defined a priori, based on their osteoporosis and/or LTF association in the published literature. The included covariables were BMI category (underweight, normal, overweight/obese [31]), diabetes mellitus, treatment with corticosteroids (ever used oral prednisone 5 mg equivalent for a cumulative duration >3 months [32] prior to LTF [matching] date), parental hip fracture history [33], smoking (current, past, never), alcohol consumption (>3 standard units daily consumption vs. less [34]), and injection drug use (IDU). We also investigated physical activity in the subset of participants with LTF after 2009, when the SHCS started recording information on physical activity. HIV-related risk factors included hepatitis C (HCV) seropositivity, CD4 nadir, and maximal log HIV viremia. ART exposures were defined a priori, based on their LTF-association in large HIV observational studies [4,5], and included tenofovir disoproxil fumarate (TDF), or boosted protease inhibitors (BPI). Individual antiretroviral agents are recorded with start and stop dates in the SHCS database. Because bone loss may be accelerated and fracture risk is increased in the first years of ART and both stabilize thereafter [35,36], we stratified ART exposures into past exposure <2 years, past exposure >2 years, current exposure <2 years, and current exposure >2 years.

### Genotyping

DNA samples were obtained from peripheral blood mononuclear cells (PBMC) and genotyped with the Global Screening Array v3.0 + MD (Illumina, San Diego, CA), or in the setting of previous SHCS genetic studies. All quality control, filtering and imputation steps prior to the merging of batches were performed separately for each batch of samples as described (S1 Appendix). For the final merged dataset used to calculate the PRS, only variants with a minor allele frequency >1% and missingness <5% were kept.

### Genome-wide polygenic risk scores

PRSs were calculated using PRSice (v2.3.5; https://www.prsice.info/). For LTF, we did not derive a de novo PRS for this analysis; rather, we applied an external, validated PRS obtained directly from the Polygenic Score Catalog (PGS000657; https://www.pgscatalog.org/), i.e., we calculated a PRS by directly applying the information on the variants included and their weights that we downloaded from the genetically predicted speed of sound (gSOS) PRS previously validated by Forgetta et al [10]. The source GWAS phenotype was broadband ultrasound attenuation and the statistical method for weight derivation was LASSO regression. As in our previous osteoporosis-PRS analysis in PWH [23], 9413 variants from the Forgetta analysis [10] were successfully matched and included. We also applied a PRS associated with longevity [27] with 4 matched variants, as we did previously for coronary artery event prediction in PWH (S1 Table) [14].

### Power calculation

In order to detect LTF odds ratios of >1.6, 255 cases and 2 controls per case would be required. As recommended, the calculations assume a correlation of exposure between pairs in the case-control set of 0.2 [37].

### Statistical analyses

Univariable and multivariable conditional logistic regression analyses were used to assess the associations of various non-genetic and genetic risk factors. We decided to stratify the genetic risk factors a priori into quintiles to facilitate visualization of potential non-linear associations with LTF. Non-genetic variables were included in the multivariable model if their association in the univariable model had a p-value <0.2. We combined all traditional and HIV-related risk factors into a single measure of non-genetic LTF risk by creating quintiles of the individually predicted LTF event probabilities from the multivariable model with the nongenetic risk factors. As cases and controls were matched on sex and age, we did not consider menopause as separate variable. Model adequacy and interactions were evaluated using Akaike and Bayesian information criteria, as well as likelihood ratio tests. Data were accessed for analyses on 28/08/2025. LTF variation explained by non-genetic risk factors and the combinations of non-genetic and genetic risk factors were documented with receiver operating characteristic (ROC) values. We used Stata/SE 18.0 (StataCorp, College Station, TX, USA).

### Sensitivity analyses

To test the robustness of the association between the gSOS-PRS and LTF, we conducted seven sensitivity analyses. Due to correlations between BMI and smoking and diabetes, we included only BMI in the main model. In the first sensitivity analysis, we reintroduced smoking and diabetes to the model. Considering potential collinearity between hepatitis C seropositivity and IDU, we conducted two separate sensitivity analyses, each excluding 1 of these 2 covariates from the multivariable model, as previously done [23]. Fourth, we excluded parental hip fracture from the model. Fifth, we adjusted the results for current and past TDF and BPI exposures as being greater or less than 6 months, as in our recent LTF time trends paper from the SHCS [24]. Sixth, we adjusted results for current and past ART exposure greater or less than 6 months rather than for TDF and BPI exposure. Lastly, to adjust for any potential population stratification, we added the first 10 principal components (PCs) to the final model.

## Results

### LTF participants

After excluding 7 cases with LTF before SHCS registration, and 8 cases without available controls, the final study population consisted of 1073 participants, i.e., 277 cases with a first LTF and 796 LTF-free controls. The number of controls per case was 1 (n = 28), 2 (n = 21), or 3 (n = 1024). Fracture sites (multiple sites possible) included spine (n = 125), femoral neck (n = 49), foot (without toes, n = 33), ribs (n = 33), lower leg (n = 42), wrist (n = 13), pelvis (n = 2), patella (n = 1), and other sites (n = 19). Participant characteristics are shown in Table 1. Observation time was similar in cases and controls. Cases were more likely to be underweight, physically inactive, current smokers, have a history of corticosteroid or injection drug use, low CD4 nadir, higher maximal HIV-viremia, and their bPI exposure was longer.

**Table 1:** Characteristics of Cases and Controls.

		Participants, Median (Interquartile Range) or No. (%)		
		All (n = 1073)	Low Trauma Fracture Cases (n = 277)	Controls (n = 796)
Male sex		734 (68.4)	186 (67.1)	548 (68.8)
Age (years)		55 (48–62)	55 (48–62)	55 (48–62)
Fracture date		--	24.10.2013 (16.12.2009–30.06.2017)	--
Duration of observation (years)^a^		13.7 (7.7–19.7)	13.6 (7.6–20.1)	13.7 (7.8–19.7)
Transmission group	Men who have Sex with Men	435 (40.5)	96 (34.7)	339 (42.6)
	Heterosexual	395 (36.8)	95 (34.3)	300 (37.7)
	Injection drug use	193 (18)	73 (26.4)	120 (15.1)
	Other/unknown	50 (4.7)	13 (4.7)	37 (4.6)
BMI	Underweight (BMI < 18.5 kg/m2)	66 (6.2)	28 (10.1)	38 (4.8)
	Normal (BMI 18.5–24.9 kg/m2)	565 (52.7)	149 (53.8)	416 (52.3)
	Overweight/Obese (BMI > 25 kg/m2)	442 (41.2)	100 (36.1)	342 (43)
Physical activity^b^	<1x/week	413/753 (54.8)	120/188 (63.8)	293/565 (51.9)
	>1x/week	340/753 (45.2)	68/188 (36.2)	272/565 (48.1)
Diabetes mellitus		98 (9.1)	20 (7.2)	78 (9.8)
Corticosteroids >3 months		73 (6.8)	35 (12.6)	38 (4.8)
Parent hip fracture		45 (4.1)	16 (5.8)	29 (3.6)
Smoking	never	352 (32.8)	80 (28.9)	272 (34.2)
	current	417 (38.9)	127 (45.8)	290 (36.4)
	past	304 (28.3)	70 (25.3)	234 (29.4)
Maximum alcohol intake^c^	none/ mild	854/971 (88)	209/253 (82.6)	645/718 (89.8)
	moderate/ heavy	122/971 (12.6)	44/253 (17.4)	78/718 (10.9)
Active injection drug use		235 (21.9)	87 (31.4)	148 (18.6)
Hepatitis C Seropositivity		270 (25.2)	99 (35.7)	171 (21.5)
CD4 at matching date (cells/μL)		561 (404–772)	490 (314–723)	582 (431–786)
CD4 nadir (cells/μL), median		162 (69–254)	130 (49–215)	174 (75–265)
CD4 nadir <200 cells/μl		660 (61.5)	191 (69)	469 (58.9)
HIV RNA max, log median		5.11 (4.61–5.6)	5.23 (4.67–5.78)	5.08 (4.57–5.54)
HIV RNA < 50 copies/mL^d^		972/1071 (90.8)	248/276 (89.9)	724/795 (91.1)
Tenofovir Disoproxil Fumarate exposure, years	All participants	2.8 (0–6.6)	2.25 (0–6.75)	2.94 (0–6.27)
	Ever exposed	4.8 (2.25-7.95)	3.74 (1.42-7.19)	4.9 (2.48-8.13)
Boosted Protease Inhibitor, exposure, years	All participants	2.59 (0–7.39)	3.55 (.04–7.71)	2.94 (0–7.27)
	Ever exposed	5.29 (2.33-9.35)	5.45 (2.13-9.86)	5.16 (2.40-9.15)

**Note.** All data shown apply to the matching date. ^a^ From registration in the SHCS until the matching date; ^b^ Information available since 12/2009, i.e., for 188 LTF cases and 565 controls; captured as number of >20 min activity units in past 6 months; ^c^ 24 LTF cases and 78 controls with missing values; ^d^ 1 LTF case and 1 control with missing values. ^e^ OR (95% CI) shown is for parent history in multivariable model without gSOS (OR for parent history was 1.88 [95% CI, 0.91–3.86; p = 0.09] in multivariable model with gSOS.

#### gSOS-PRS and longevity-PRS in cases and controls.

**Fig 1:**
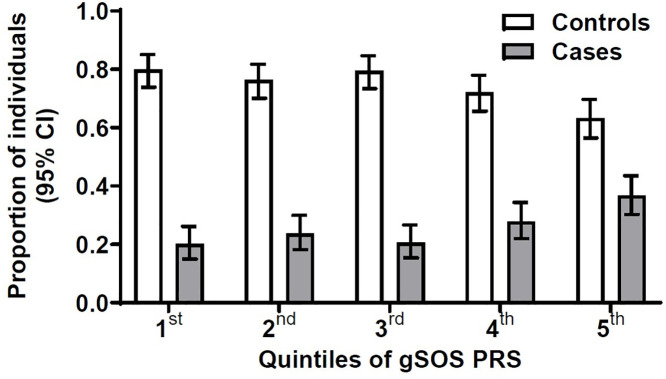
Distribution of gSOS-polygenic risk score in 796 controls without LTF (white bars) and in 277 cases with a first LTF (gray bars). We divided study participants into 5 quintiles according to their individual gSOS-polygenic risk score. Shown here are the number, percentage and 95% confidence intervals of participants in each quintile. A: Distribution of LTF cases and controls according to quintiles of gSOS-PRS. There were 43 (20.1%) cases vs. 171 (79.9%) controls in the 1^st^ (most favorable) quintile, 51 (23.7%) vs. 164 (76.3%) in the 2^nd^ quintile, 44 (20.6%) vs. 170 (79.4%) in the 3^rd^ quintile, 60 (27.9%) vs. 155 (72.1%) in the 4^th^ quintile, and 79 (36.7%) vs. 136 (63.3%) in the 5^th^ (most unfavorable) quintile. **Abbreviations:** LTF, low trauma fracture; CI, confidence interval; PRS, polygenic risk score.

Cases had higher median gSOS-PRS values and thus higher genetic LTF risk than controls (p < 0.01; S2 Table). The distribution of cases among the gSOS-PRS quintiles was asymmetric, i.e., skewed towards the 5th (most unfavorable) vs. the other quintiles of genetic risk (Fig 1). For longevity-PRS, there was no evidence for longevity-PRS values being different among cases and controls (p = 0.39; S2 Table) and cases and controls were distributed similarly across longevity-PRS quintiles (S1 Fig.).

### Univariable analyses

#### Association of LTF with PRSs.

**Fig 2:**
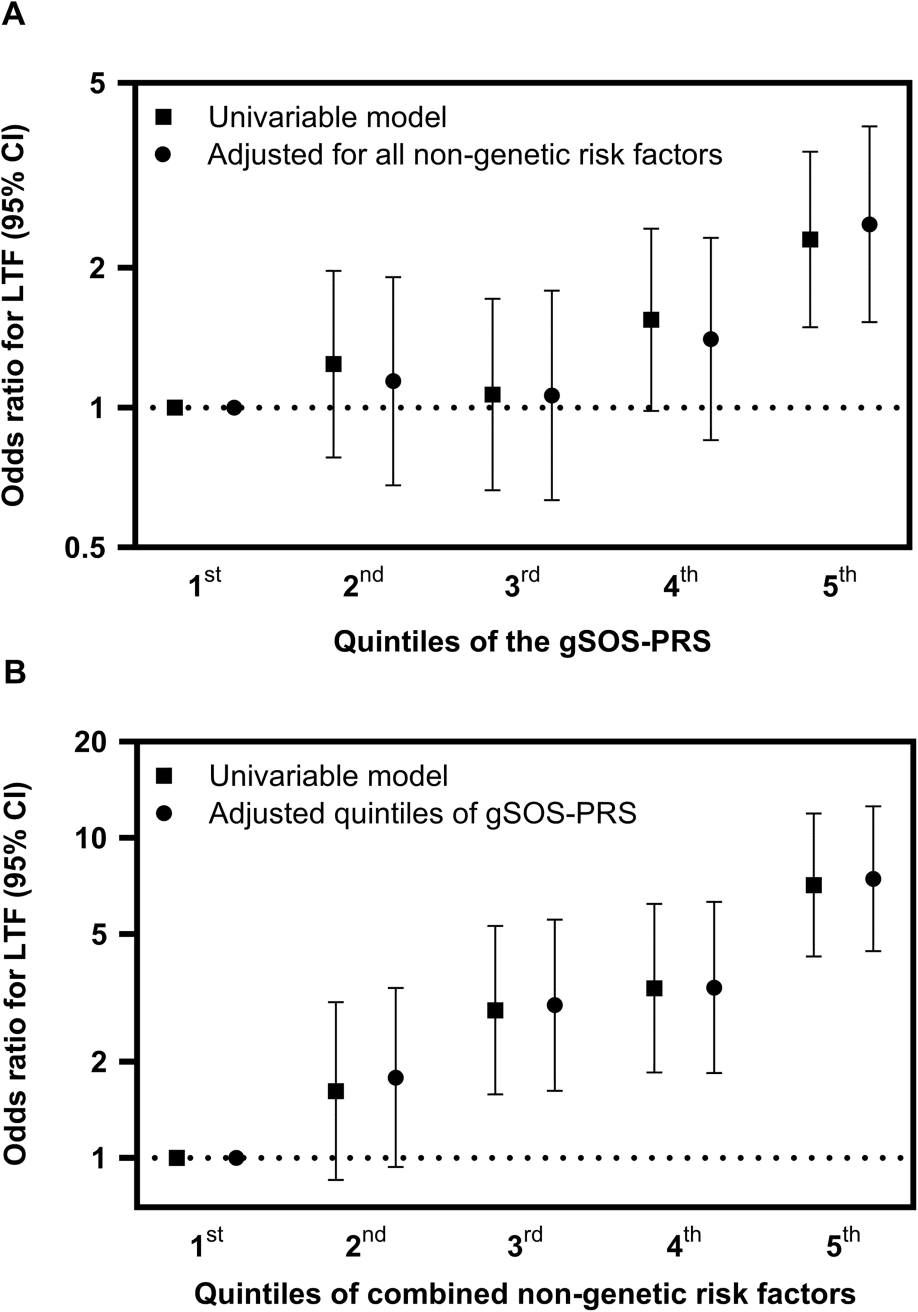
Odds Ratios for Low Trauma Fractures according to gSOS Polygenic Risk Score, and According to Combined Non-Genetic Risk Factors. **A)** Odds ratios (ORs) for low trauma fractures (LTF, with 95% confidence intervals [CIs]), according gSOS-polygenic risk score, unadjusted and adjusted for combined non-genetic risk factors (listed in the Methods and in Table 1). Results show univariable and multivariable conditional logistic regression of associations of gSOS-PRS with LTF for 277 LTF cases and 796 LTF-free controls. Compared with the first (most favorable) quintile of genetic (gSOS-PRS) risk, participants in the fifth (most unfavorable) quintiles had significantly increased LTF-OR that remained similar when adjusted for combined non-genetic risk factors. **B)** Odds ratios and 95% CIs for low trauma fractures according to quintiles of combined non-genetic risk factors. Results were similar with and without adjustment for gSOS-PRS. For exact odds ratios, 95% CIs, and p-values, refer to S2 Table. Abbreviations: LTF, low trauma fracture; CI, confidence interval; PRS, polygenic risk score.

LTF were significantly associated with gSOS-PRS in continuous analysis (p < 0.01; S2 Appendix). When compared with the first gSOS-PRS quintile (most favorable genetic background), participants in fifth quintile (most unfavorable genetic background) had increased LTF-OR (OR=2.30 [95% CI, 1.49–3.56]), but participants in the second, third, and fourth quintile did not have increased LTF-OR, with ORs of 1.24 (95% CI,.78–1.97), 1.07 (.66–1.72), 1.55 (.98–2.43), respectively (Fig 2A, S3 Table). LTF risk was not associated with longevity-PRS in univariable analysis (p = 0.49; S2 Appendix and S4 Table), therefore longevity-PRS was not included in multivariable model.

#### LTF Probability According to Non-Genetic Risk Factors, Univariable analysis without correction for gSOS-PRS.

LTF were associated with injection drug use, underweight BMI, corticosteroid use, current smoking, hepatitis C co-infection, and inversely associated with physical activity (S5 Table). Compared with participants in the first (most favorable) quintile of *combined* non-genetic LTF risk, those in the second, third, fourth, and fifth (most unfavorable) quintiles had univariable LTF-ORs of 1.76 (95% confidence interval [CI],.9–3.42), 4.43 (2.4–8.17), 5.18 (2.79–9.65), and 7.36 (4.28–12.63), respectively (Fig 2B, S3 Table).

### Multivariable analyses

#### LTF Probability According to gSOS-PRS.

Compared to the first gSOS-PRS quintile, participants in the second, third, fourth, and fifth quintiles had LTF-OR=1.20 (0.73–1.98), 1.00 (0.59–1.68), 1.35 (0.82–2.22), and 2.30 (1.43–3.72), respectively (Fig 2A; S3 Table).

#### LTF probability according to non-genetic risk factors.

LTFs remained associated with corticosteroid use, hepatitis C co-infection, maximal HIV RNA level, and inversely associated with physical activity (S5 Table). LTF probability remained significantly associated with combined non-genetic risk factors, after adjustment for gSOS-PRS (Fig 2B). Compared with participants in the first (most favorable) quintile of combined non-genetic LTF risk, those in the second, third, fourth, and fifth (most unfavorable) quintiles had multivariable LTF-ORs of 1.79 (95% CI,.92–3.51), 4.49 (2.41–8.34), 5.02 (2.67–9.43), and 7.42 (4.3–12.82), respectively (P < .001; Fig 2B, S3 Table).

#### Sensitivity analyses.

Results remained similar in all sensitivity analyses, including when we added diabetes and smoking back to the model (S6 Table); when we included only IDU but not hepatitis C in the model (S7 Table); when we included only hepatitis C but not IDU in the model (S8 Table); when we removed parental hip fracture history from the model (S9 Table), and when we included the first 10 principal components in the model (S10 Table).

#### LTF variability explained by non-genetic risk factors and gSOS-PRS.

The area under the ROC curve (ROC AUC) for non-genetic risk factors was 0.736. The ROC AUC increased significantly (p = 0.0023) to 0.767 when adding gSOS-PRS and principal components 1–10.

## Discussion

The main finding of this study is that an unfavorable genetic background increases LTF risk around 2.3-fold in 20% of PWH, i.e., those in the top (most unfavorable) vs. the bottom (most favorable) gSOS-PRS quintile. Results were similar when we adjusted for traditional and HIV-related LTF risk factors and in multiple sensitivity analyses, suggesting the effect of gSOS-PRS is robust. In addition, we provide a combined estimate of the impact of non-genetic LTF risk factors and show that the highest non-genetic risk category was associated with a 7.4-fold increased LTF risk. Thus, while non-genetic risk factors explained a larger proportion of LTF than genetic background, non-genetic and genetic models independently predicted LTF.

Our results are likely to be relevant for future research and future HIV clinical care. It is likely that genetic testing will enter routine medical care in the next 10 years. That is, PRS for various hard end points relevant to aging people with HIV (diabetes, chronic kidney disease, steatotic liver disease, obesity, cardiovascular endpoints, osteoporosis, low trauma fractures etc.) might then be measured at an early stage in HIV care. For example, knowledge of an unfavorable bone mineral density-associated and LTF-associated PRS (top quintile of the gSOS-PRS) may encourage early attention to osteoporosis screening in at-risk individuals, encourage them to pursue other prevention measures including smoking cessation, a physically active lifestyle, supplementation of calcium and vitamin D, and finally, draw the clinician’s attention to avoidance of antiretrovirals with a potentially adverse LTF risk profile. On the other hand, while a longevity-associated PRS was associated with coronary events in our previous study of Swiss PWH [14), we were unable to document an association of longevity-associated PRS with LTF. This likely confirms prior knowledge that longevity is more closely linked to coronary events than to LTF.

Our present study including 277 people with HIV with LTF and 796 fracture-free controls expands on our previous report showing that the gSOS-PRS was independently associated with DXA-defined osteoporosis in 438 people with HIV in Switzerland [23]. In PWH, the effect size of an unfavorable gSOS-PRS (top quintile) regarding osteoporosis may be larger than the effect size regarding LTF, i.e., approx. 4-fold osteoporosis risk increase in our previous report [23] vs. approx. 2.3-fold LTF risk increase in the present study. Each of these effect sizes suggest that the effect size of gSOS-PRS on both osteoporosis and LTF is clinically relevant. Moreover, LTF is a hard clinical endpoint that is associated with substantial morbidity (hospital admissions, surgical treatment, lost productivity) and cost to health systems [38,39]. Importantly, a large proportion of LTF [40] occurs in persons without any documented osteoporosis, suggesting considerable clinical interest in genetic prediction of LTF [41].

Our results appear to stand in contrast to our previous small LTF study which was unable to document any significant LTF-association with 6 selected SNPs [25]. This is likely explained by the application of an individual PRS in the present study. Moreover, here we evaluated 277 PWH with LTF rather than previously only 103 PWH with LTF, i.e., the study population was nearly three times as large as in our previous report [25]. Additional strengths of the present study include the exploitation of prospectively recorded rich clinical information from longitudinally followed participants of the well-established Swiss HIV Cohort Study.

While parent history of hip fracture did not predict LTF, the gSOS-PRS independently did predict LTF, and the gSOS-PRS-effect was similar with or without adjustment for parent hip fracture history. This finding may have a number of explanations. First, participants may underreport or incorrectly report parent hip fracture history. Second, gSOS-PRS and parent history likely capture independent, complementary effects on LTF in PWH, similar to findings by others and us regarding family history, coronary artery disease-associated PRSs and coronary events [42–44]. Third, family history may reflect not only genetic background, but also environmental, social, and lifestyle factors shared among family members, as previously discussed [42]. Last, in the general population, parent history of hip fracture may increase fracture risk at the hip but the risk increase at other sites including vertebral and other fractures is small [33].

In this study and in our previous report on LTF time trends in the SHCS [24], TDF therapy was not associated with significantly increased LTF risk. This may appear surprising, but the literature on the associations of TDF with fracture risk is mixed. Previous reports in HIV show a 12% risk increase per year TDF exposure in the large Veterans Affairs Clinical Case Registry (951 study participants with LTF) (4), but no significant risk increase with TDF in the Women’s Interagency HIV Study (106 women with LTF) [45], the small female Alfred Hospital/Australia HIV study (61 participants with LTF) [46], and the EuroSIDA study (496 participants with LTF) (5), and even a reduced fracture risk with TDF in the Ingenix Impact National Benchmark Database (2477 participants with fractures) [47]. Importantly, as per international HIV treatment guidelines, clinicians may not prescribe TDF in PWH with increased fracture risk and have been deprescribing TDF and bPI in recent years [24,48]. The motivations of clinicians have included declining renal function, concerns about osteoporosis and a greater emphasis in recent years on bone health in HIV in general, plus the availability, efficacy and tolerability of HIV integrase inhibitors. These prescribing changes have likely made it more difficult to capture any bone-toxic effects of TDF and bPIs in epidemiological studies. Consistent with previous reports recording acceleration of bone mineral density loss and increased fracture risk in the first two years of ART [35,36], we show a trend and a significantly increased LTF risk in the first two years after TDF and boosted PI start, respectively.

Strengths of our genetic results include rigorous quality control, correction for residual population stratification, exclusion of population outliers, plus the independent association of gSOS-PRS with LTF persisting after adjusting for relevant non-genetic LTF-risk factors. These risk factors were selected based on published literature in order to obtain a strong traditional and HIV-related LTF risk model, and to compare the effects of non-genetic and genetic LTF risk factors. In our dataset, the non-genetic LTF risk factors showed associations with LTF as expected. It is important to note, however, that we did not perform any causal modeling of individual non-genetic LTF risk factors and their effect sizes should therefore be interpreted with caution. Additional limitations of our results include the lack of systematic bone mineral density data, no data on falls, limited availability of physical activity data (since 2009), and relevant non-ART medication including vitamin D, calcium, and bisphosphonates (data only available since 2015), limited information on socioeconomic and lifestyle data, and insufficient details (e.g., diagnosis date, anti-inflammatory treatment history) regarding chronic inflammatory conditions such as rheumatoid arthritis. Fracture risk increases with prolonged corticosteroid use and fracture risk decreases back to baseline risk within 6–12 months after discontinuing corticosteroids. [49–51] However, we were unable to assess the exact dates and therefore, recency, of corticosteroid use in more than half of our participants who had LTFs before 2015. Because most GWAS regarding LTF have been performed in populations of European ancestry, our study was restricted to participants of European ancestry, limiting generalizability. Given the predominantly male, middle-aged composition of the study population, generalizability of our results to other populations is limited. It is beyond the scope of our study to assess the clinical value of genetic testing – prospective studies are needed for this. An additional likely limitation is variability in fracture detection across clinical sites.

In conclusion, low trauma fractures are of considerable relevance in PWH—for whom osteoporosis risk factors and the cumulative effects of certain ART agents may be prevalent and aging may be accentuated or even accelerated [52,53]. In our study of PWH in Switzerland, an unfavorable genetic background, as captured by being in the top quintile of the bone mineral density-associated gSOS-PRS, independently increased LTF risk more than 2-fold, compared to the bottom gSOS-PRS quintile, when adjusted for multiple traditional and HIV-related risk factors.

## Supporting information

S1 Appendix Supplementary Methods. (DOCX)

S2 Appendix Supplementary Results. (DOCX)

S1 Table Single nucleotide polymorphisms included in the Longevity PRS. (DOCX)

S2 Table Distribution of gSOS-polygenic risk score in 796 controls without LTF and in 277 cases with a first LTF PRS and longevity-polygenic risk score in 757 controls without LTF and in 275 cases with a first LTF. (DOCX)

S3 Table Low Trauma Fracture Odds Ratio according to Non-Genetic Risk Factors and gSOS-PRS, Univariable and Multivariable Analysis. (DOCX)

S4 Table Low Trauma Fracture Odds Ratios and 95% Confidence Intervals According to Longevity-PRS, Univariable Analysis. (DOCX)

S5 Table Low Trauma Fracture Odds Ratio According to Non-Genetic Risk Factors: Univariable and Multivariable Analysis Without gSOS Polygenic Risk Score. (DOCX)

S6 Table Sensitivity Analysis: LTF Odds Ratio Including not only BMI, but additionally Diabetes mellitus and Smoking in the Multivariable Model. (DOCX)

S7 Table Sensitivity Analysis: LTF Odds Ratio Including only Injection Drug Use, but not Hepatitis C in the Multivariable Model. (DOCX)

S8 Table Sensitivity Analysis: LTF Odds Ratio Including only Hepatitis C, but not Injection Drug Use in the Multivariable Model. (DOCX)

S9 Table Sensitivity Analysis: LTF Odds Ratio Excluding Parental Hip Fracture History from the Multivariable Model. (DOCX)

S10 Table Sensitivity Analysis: LTF Odds Ratio Including the first 10 Principal Components in the Multivariable Model. (DOCX)

S1 Fig Distribution of longevity-polygenic risk score in 796 controls without LTF and in 277 cases with a first LTF. (DOCX)
