# Supplementary material for: Association of a polygenic risk score with low trauma fractures in people with HIV – The swiss HIV cohort study
Source: PLoS One. 2026 Feb 11;21(2):e0342748. doi: 10.1371/journal.pone.0342748 (PMC12893606; doi:10.1371/journal.pone.0342748)
Supplement: S1 Appendix — (DOCX) [file pone.0342748.s001.docx]

**S1 Appendix. Supplementary Methods.**

**Genotyping, Quality Control.** For each genotyping batch, samples and SNPs were removed if having an excessive genotype missingness (>10%) or if the minor allele frequency deviated more than 20% from that of the 1000 Genomes Project Phase 3 EUR reference panel. Missing genotypes were imputed using positional Burrows-Wheeler transformation (PBWT) [2], at the Sanger Imputation Service,[1] using the 1000 Genomes Project Phase 3 panel as reference. Phasing was performed using EAGLE2 [3]. Only high-quality SNPs with an imputation information score (INFO > 0.8) were retained following the imputation, after which the genotyping batches were combined. Principal components and population structure was calculated with EIGENSTRAT (v6.1.4)[4], together with the HapMap3 reference panel [5]. For the subsequent genetic risk score, only individuals clustering with the European HapMap3 samples were included. The cohort was furthermore screened with KING (v2.1.3)[6] to ensure that no cryptic related or duplicate samples were included. Lastly, samples and SNPs with excessive missingness (above 10%), low minor allele frequency (below 1%) or excessive deviation from Hardy-Weinberg Equilibrium (P_HWE_ < 1e-6) were removed prior to calculating the genetic risk scores.

**Supplementary Bibliography**

1. Durbin R. Efficient haplotype matching and storage using the positional Burrows-Wheeler transform (PBWT). *Bioinformatics.* 2014; 30:1266–1272.

2. McCarthy S, Das S, Kretzschmar W, et al. A reference panel of 64,976 haplotypes for genotype imputation. *Nat Genet.* 2016; 48:1279–1283.

3. Loh P-R, Danecek P, Palamara PF, et al. Reference-based phasing using the Haplotype Reference Consortium panel. *Nat Genet.* 2016; 48:1443–1448.

4. Price AL, Patterson NJ, Plenge RM, Weinblatt ME, Shadick NA, Reich D. Principal components analysis corrects for stratification in genome-wide association studies. *Nat Genet.* 2006; 38:904–909.

5. The International HapMap 3 Consortium. Integrating common and rare genetic variation in diverse human populations. *Nature.* 2010; 467:52–58.

6. Manichaikul A, Mychaleckyj JC, Rich SS, Daly K, Sale M, Chen W-M. Robust relationship inference in genome-wide association studies. *Bioinformatics.* 2010; 26:2867–2873.
