# Supplementary material for: Association of a polygenic risk score with low trauma fractures in people with HIV – The swiss HIV cohort study
Source: PLoS One. 2026 Feb 11;21(2):e0342748. doi: 10.1371/journal.pone.0342748 (PMC12893606; doi:10.1371/journal.pone.0342748)
Supplement: S2 Appendix — (DOCX) [file pone.0342748.s002.docx]

**S2 Appendix. Supplementary Results.**

**LTF** **Probability According to gSOS-PRS and longevity PRS, Univariable analysis.** Per standard deviation increase (less favorable) in the gSOS PRS, LTF odds ratio was 1.19 (95% confidence interval, 1.10-1.29). Per standard deviation increase (less favorable) in the longevity PRS, LTF odds ratio was 3.44 (95% confidence interval, 0.11-111.53).

**LTF** **Probability According to gSOS-PRS, Multivariable Analysis.** Per standard deviation increase (less favorable) in the gSOS PRS, LTF odds ratio in the final multivariable model was 1.17 (95% confidence interval, 1.08-1.28).
