# Supplementary material for: Association of a polygenic risk score with low trauma fractures in people with HIV – The swiss HIV cohort study
Source: PLoS One. 2026 Feb 11;21(2):e0342748. doi: 10.1371/journal.pone.0342748 (PMC12893606; doi:10.1371/journal.pone.0342748)
Supplement: S1 Table — (DOCX) [file pone.0342748.s003.docx]

**S1 Table. Single nucleotide polymorphisms included in the Longevity PRS**

| **rsID** | **Gene** | **effect_allele** | **reference_allele** | **Beta_GWAS** | **SE_GWAS** |
| --- | --- | --- | --- | --- | --- |
| rs7412 | *APOE* | C | T | .2452 | .0367 |
| rs6859 | *NECTIN2* | G | A | -.1124 | .02 |
| rs429358 | *APOE* | T | C | .5098 | .0322 |
| rs405509 | *APOE* | G | T | -.1299 | .0199 |
