# Supplementary material for: Association of a polygenic risk score with low trauma fractures in people with HIV – The swiss HIV cohort study
Source: PLoS One. 2026 Feb 11;21(2):e0342748. doi: 10.1371/journal.pone.0342748 (PMC12893606; doi:10.1371/journal.pone.0342748)
Supplement: S2 Table — (DOCX) [file pone.0342748.s004.docx]

**S2 Table. Distribution of gSOS-polygenic risk score in 796 controls without LTF and in 277 cases with a first LTF PRS and longevity-polygenic risk score in 757 controls without LTF and in 275 cases with a first LTF.**

|  | **Quintiles of gSOS-Polygenic Risk Score** | | **Quintiles of longevity-Polygenic Risk Score** | |
| --- | --- | --- | --- | --- |
|  | **Cases**  **No. (%)** | **Controls**  **No. (%)** | **Cases**  **No. (%)** | **Controls**  **No. (%)** |
| 1^st^ Quintile | 43 (20) | 171 (79.9) | 53 (26) | 151 (74) |
| 2^nd^ Quintile | 51 (23.7) | 164 (76.9) | 41 (29.3) | 99 (70.7) |
| 3^rd^ Quintile | 44 (20.6) | 170 (79.4) | 53 (21.8) | 190 (78.2) |
| 4^th^ Quintile | 60 (27.9) | 155 (72.1) | 45 (29.8) | 106 (70.2) |
| 5^th^ Quintile | 79 (36.7) | 136 (63.3) | 83 (28.2) | 211 (71.8) |
