# Supplementary material for: Association of a polygenic risk score with low trauma fractures in people with HIV – The swiss HIV cohort study
Source: PLoS One. 2026 Feb 11;21(2):e0342748. doi: 10.1371/journal.pone.0342748 (PMC12893606; doi:10.1371/journal.pone.0342748)
Supplement: S3 Table — (DOCX) [file pone.0342748.s005.docx]

**S3 Table. Low Trauma Fracture Odds Ratio (OR) according to Non-Genetic Risk Factors and gSOS-PRS, Univariable and Multivariable Analysis.**

|  | **Quintiles of gSOS-Polygenic Risk Score** | | **Quintiles of Combined Non-Genetic Risk Factors**  **(=Traditional plus HIV-related Risk Factors)** | |
| --- | --- | --- | --- | --- |
|  | **Univariable Analysis,**  **OR (95% CI);**  **P-Value** | **Multivariable Analysis Main Model, adjusted for Non-Genetic Risk Factors, OR (95% CI); P-Value** | **Univariable Analysis OR (95% CI); P-Value** | **Multivariable Analysis adjusted for gSOS-PRS, OR (95% CI); P-Value** |
| 1^st^ Quintile | (reference) | (reference) | (reference) | (reference) |
| 2^nd^ Quintile | 1.24 (.78–1.97);  .36 | 1.2 (.73–1.98);  .48 | 1.76 (.9–3.42);  .10 | 1.79 (.92–3.51);  .09 |
| 3^rd^ Quintile | 1.07 (.66–1.72);  .79 | 1 (.59–1.68);  .99 | 4.43 (2.4–8.17);  <.001 | 4.49 (2.41–8.34);  <.001 |
| 4^th^ Quintile | 1.55 (.98–2.43);  .06 | 1.35 (.82–2.22);  .24 | 5.18 (2.79–9.65);  <.001 | 5.02 (2.67–9.43);  <.001 |
| 5^th^ Quintile | 2.3 (1.49–3.56);   <.001 | 2.3 (1.43–3.72);  .001 | 7.36 (4.28–12.63);  <.001 | 7.42 (4.3–12.82);   <.001 |

**Note.** The odds ratios and 95% confidence intervals shown here are also shown in **Figure 2.**

**Abbreviations.** CI, confidence interval; OR, odds ratio; PRS, polygenic risk score
