# Supplementary material for: Association of a polygenic risk score with low trauma fractures in people with HIV – The swiss HIV cohort study
Source: PLoS One. 2026 Feb 11;21(2):e0342748. doi: 10.1371/journal.pone.0342748 (PMC12893606; doi:10.1371/journal.pone.0342748)
Supplement: S4 Table — (DOCX) [file pone.0342748.s006.docx]

**S4 Table. Low Trauma Fracture Odds Ratios and 95% Confidence Intervals According to Longevity-PRS, Univariable Analysis.**

|  | **Quintiles of Longevity-PRS** |
| --- | --- |
|  | ***Univariable Analysis***  Odds ratio, 95% confidence interval; P-value |
| 1^st^ Quintile | (reference) |
| 2^nd^ Quintile | 1.16 (.71–1.88); .56 |
| 3^rd^ Quintile | .78 (.5–1.22); .28 |
| 4^th^ Quintile | 1.26 (.77–2.06); .35 |
| 5^th^ Quintile | 1.14 (.75–1.73); .53 |

**Abbreviations.** PRS, polygenic risk score**.**
