# Supplementary material for: Association of a polygenic risk score with low trauma fractures in people with HIV – The swiss HIV cohort study
Source: PLoS One. 2026 Feb 11;21(2):e0342748. doi: 10.1371/journal.pone.0342748 (PMC12893606; doi:10.1371/journal.pone.0342748)
Supplement: S5 Table — (DOCX) [file pone.0342748.s007.docx]

**S5 Table.** **Low Trauma Fracture Odds Ratio According to Non-Genetic Risk Factors: Univariable and Multivariable Analysis Without gSOS Polygenic Risk Score.**

|  | | ***Univariable Analysis***  Odds ratio, 95% confidence interval;  P-value | ***Multivariable Analysis***  Odds ratio, 95% confidence interval;  P-value |
| --- | --- | --- | --- |
| Transmission group | Men who have Sex with Men | 1 [Reference] | 1 [Reference] |
|  | Heterosexual | 1.16 (.78–1.73);  .46 | 1.05 (.68–1.61);  .84 |
|  | Injection drug use | 2.84 (1.81–4.45);  <.001 | 1.53 (0.68–1.61);  .20 |
|  | Other/unknown | 1.24 (.60–2.54);  .57 | 1.21 (.56–2.65);  .63 |
| BMI | Underweight  (BMI <18.5 kg/m2) | 2.06 (1.21–3.50);  .008 | 1.60 (.88–2.91);  .12 |
|  | Normal  (BMI 18.5-24.9 kg/m2) | 1 [Reference] | 1 [Reference] |
|  | Overweight/Obese  (BMI >25 kg/m2) | .78 (.57–1.07);  .12 | .74 (.53-–1.05);  .10 |
| Physical activity ^b^ | <1x/week | 1 [Reference] | 1 [Reference] |
|  | >1x/week | .6 (.42–.84);  0.003 | .69 (.47–1.00);  .05 |
| Diabetes mellitus |  | .70 (.41–1.19);  .19 | -- |
| Corticosteroids >3 months |  | 2.83 (1.75–4.59);  <.001 | 3.12 (1.81–5.39);  <.001 |
| Parent hip fracture |  | 1.46 (.92–2.30);  .10 ^e^ | 1.53 (.89–2.63);  .12 ^e^ |
| Smoking | never | 1 [Reference] | -- |
|  | current | 1.67 (1.17–2.38);  .005 | -- |
|  | past | 1.05 (.71–1.53);  .815 | -- |
| Maximum alcohol intake ^c^ | none / mild | 1 [Reference] | 1 [Reference] |
|  | moderate / heavy | 1.28 (.96–1.71);  .09 | 1.34 (.97–1.86);  .08 |
| Active injection drug use |  | -- | -- |
| Hepatitis C Seropositivity |  | 2.58 (1.81–3.68);  <.001 | 1.91 (1.13–3.23);  .02 |
| CD4 at matching date (cells/μL) |  | -- | -- |
| CD4 nadir (cells/μL), median |  | -- | -- |
| CD4 nadir <200 cells/μl |  | 1.57 (1.15–2.12);  .004 | 1.23 (.87–1.74);  .25 |
| HIV RNA max, log median |  | 1.19 (1.01–1.40);  .03 | 1.25 (1.03–1.50);  .02 |
| HIV RNA<50 copies/mL ^d^ |  | -- | -- |
| Tenofovir Disoproxil Fumarate exposure, years | All participants | Past exposure <2 years:  1.52 (.93–2.49); .09  Past exposure 2+ years:  0.99 (.63-1.57); .96  Current exposure <2 years:  2.53 (1.39-4.59); .002  Current exposure 2+ years:  0.94 (0.63-1.39); .74 | Past exposure <2 years:  1.39 (.80–2.41); .25  Past exposure 2+ years:  0.71 (.43-1.19); .20  Current exposure <2 years:  1.80 (0.90-3.62); .10  Current exposure 2+ years:  0.74 (0.47-1.16); .19 |
|  | Ever exposed |  |  |
| Boosted Protease Inhibitor, exposure, years | All participants | Past exposure <2 years:  1.15 (.70–1.89); .57  Past exposure 2+ years:  1.30 (.84-2.04); .24  Current exposure <2 years:  4.07 (1.86-8.88); <0.001  Current exposure 2+ years:  1.77 (1.21-2.59); .003 | Past exposure <2 years:  1.06 (0.62–1.81); .84  Past exposure 2+ years:  1.07 (.65-1.77); .78  Current exposure <2 years:  3.08 (1.24-7.68); .016  Current exposure 2+ years:  1.52 (0.97-2.38); .069 |
|  | Ever exposed |  |  |
