# Supplementary material for: Association of a polygenic risk score with low trauma fractures in people with HIV – The swiss HIV cohort study
Source: PLoS One. 2026 Feb 11;21(2):e0342748. doi: 10.1371/journal.pone.0342748 (PMC12893606; doi:10.1371/journal.pone.0342748)
Supplement: S6 Table — (DOCX) [file pone.0342748.s008.docx]

**S6 Table. Sensitivity Analysis: LTF Odds Ratio (OR) Including not only BMI, but additionally Diabetes mellitus and Smoking in the Multivariable Model.**

|  | **gSOS-Polygenic Risk Score** |
| --- | --- |
|  | ***Multivariable Analysis***  ***adjusted for all non-genetic risk factors*** OR (95% CI); P-Value |
| 1^st^ Quintile | (reference) |
| 2^nd^ Quintile | 1.33 (.81–2.18); .27 |
| 3^rd^ Quintile | 1.09 (.66–1.82); .73 |
| 4^th^ Quintile | 1.4 (.86–2.28); .18 |
| 5^th^ Quintile | 2.33 (1.44–3.68); <.001 |

**Abbreviations.** CI, confidence interval; OR, odds ratio; PRS, polygenic risk score.
