# Supplementary material for: Association of a polygenic risk score with low trauma fractures in people with HIV – The swiss HIV cohort study
Source: PLoS One. 2026 Feb 11;21(2):e0342748. doi: 10.1371/journal.pone.0342748 (PMC12893606; doi:10.1371/journal.pone.0342748)
Supplement: S8 Table — (DOCX) [file pone.0342748.s010.docx]

**S8 Table. Sensitivity Analysis: LTF Odds Ratio (OR) Including only Hepatitis C, but not Injection Drug Use in the Multivariable Model.**

|  | **gSOS-Polygenic Risk Score** |
| --- | --- |
|  | ***Multivariable Analysis***  ***adjusted for all non-genetic risk factors* OR (95% CI); P Value** |
| 1^st^ Quintile | (reference) |
| 2^nd^ Quintile | 1.19 (.72–1.96); .5 |
| 3^rd^ Quintile | .98 (.59–1.64); .94 |
| 4^th^ Quintile | 1.35 (.82–2.22); .24 |
| 5^th^ Quintile | 2.33 (1.44–3.76); .001 |

**Abbreviations.** CI, confidence interval; OR, odds ratio; PRS, polygenic risk score.
