# Supplementary material for: Association of a polygenic risk score with low trauma fractures in people with HIV – The swiss HIV cohort study
Source: PLoS One. 2026 Feb 11;21(2):e0342748. doi: 10.1371/journal.pone.0342748 (PMC12893606; doi:10.1371/journal.pone.0342748)
Supplement: S9 Table — (DOCX) [file pone.0342748.s011.docx]

**S9 Table. Sensitivity Analysis: LTF Odds Ratio (OR) Excluding Parental Hip Fracture History from the Multivariable Model.**

|  | **gSOS-Polygenic Risk Score** |
| --- | --- |
|  | ***Multivariable Analysis***  ***adjusted for all non-genetic risk factors* OR (95% CI); P Value** |
| 1^st^ Quintile | (reference) |
| 2^nd^ Quintile | 1.21 (.73–2); .45 |
| 3^rd^ Quintile | 1 (.6–1.68); .99 |
| 4^th^ Quintile | 1.34 (.81–2.19); .25 |
| 5^th^ Quintile | 2.27 (1.44–3.65); .001 |

**Abbreviations.** CI, confidence interval; OR, odds ratio; PRS, polygenic risk score.
