# Supplementary material for: Association of a polygenic risk score with low trauma fractures in people with HIV – The swiss HIV cohort study
Source: PLoS One. 2026 Feb 11;21(2):e0342748. doi: 10.1371/journal.pone.0342748 (PMC12893606; doi:10.1371/journal.pone.0342748)
Supplement: S10 Table — (DOCX) [file pone.0342748.s012.docx]

**S10 Table. Sensitivity Analysis: LTF Odds Ratio Including the first 10 Principal Components in the Multivariable Model.**

|  | **gSOS-Polygenic Risk Score** |
| --- | --- |
|  | **First 10 principal components OR (95% CI); P Value** |
| pc1 | 2.68e+08 (8.59e-26—8.35e+41); .622 |
| pc2 | 4.266455 (2.37e-14 7.68e+14); .931 |
| pc3 | 1.04e-10 (1.37e-28 7.85e+07); .274 |
| pc4 | 1.57e+16 (5.35e-07 4.60e+38); .158 |
| pc5 | 1.67e+07 (2.11e-06 1.33e+20); .272 |
| pc6 | .1765356 (1.66e-07 187664.4); .806 |
| pc7 | 1.52e+09 (2.02e-07 1.14e+25); .257 |
| pc8 | .0645897 (7.57e-15 5.51e+11); .857 |
| pc9 | .0045029 (1.05e-11 1933222); .594 |
| pc10 | .0010055 (2.69e-11 37610.92); .438 |
|  | ***Multivariable Analysis***  ***adjusted for all non-genetic risk factors and first 10 principal components* OR (95% CI); P Value** |
| 1^st^ Quintile | (reference) |
| 2^nd^ Quintile | 1.15 (.68–1.96); .6 |
| 3^rd^ Quintile | 1.16 (.68–1.95); .59 |
| 4^th^ Quintile | 1.42 (.85–2.37); .18 |
| 5^th^ Quintile | 2.56 (1.56–4.18); <.001 |

**Abbreviations.** CI, confidence interval; OR, odds ratio; pc, principal component; PRS, polygenic risk score.
