# Supplementary material for: Association of a polygenic risk score with low trauma fractures in people with HIV – The swiss HIV cohort study
Source: PLoS One. 2026 Feb 11;21(2):e0342748. doi: 10.1371/journal.pone.0342748 (PMC12893606; doi:10.1371/journal.pone.0342748)
Supplement: S1 Fig — (DOCX) [file pone.0342748.s013.docx]

**S1 Figure. Distribution of longevity-polygenic risk score in 796 controls without LTF (white bars) and in 277 cases with a first LTF (gray bars).**

We divided study participants into 5 quintiles according to their individual longevity-polygenic risk score. Shown here are the number, percentage and 95% confidence intervals of participants in each quintile.


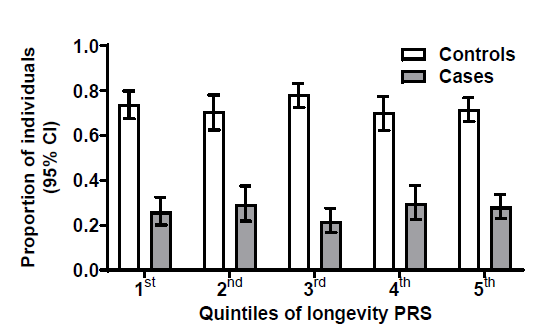


**A:** **Distribution of LTF cases and controls according to quintiles of longevity-PRS**. There were 53 (26%) cases vs. 151 (74%) controls in the 1^st^ (most favorable) quintile, 41 (29.3%) vs. 99 (70.7%) in the 2^nd^ quintile, 53 (21.8%) vs. 190 (78.2%) in the 3^rd^ quintile, 45 (29.8%) vs. 106 (70.2%) in the 4^th^ quintile, and 83 (28.2%) vs. 211 (71.8%) in the 5^th^ (most unfavorable) quintile.

**Abbreviations**: LTF, low trauma fracture; CI, confidence interval; PRS, polygenic risk score.
